# BCS2.0: a capture sequencing platform for rapid differential diagnosis of bacterial infections and antimicrobial resistance

**DOI:** 10.3389/fmicb.2026.1802485

**Published:** 2026-04-20

**Authors:** Amit Ranjan, Cheng Guo, Thomas Briese, William Donovan, Vishal Kapoor, Alamelu Chandrasekaran, Mahesh M. Mansukhani, Gregory J. Berry, W. Ian Lipkin

**Affiliations:** 1 Center for Infection and Immunity, Mailman School of Public Health, Columbia University, New York, NY, United States; 2 School of Public Health, Sun Yat-Sen University, Guangzhou, China; 3 Department of Epidemiology, Mailman School of Public Health, Columbia University, New York, NY, United States; 4 Department of Pathology and Cell Biology, Vagelos College of Physicians and Surgeons, Columbia University, New York, NY, United States; 5 Center for Advanced Laboratory Medicine, Vagelos College of Physicians and Surgeons, Columbia University, New York, NY, United States; 6 Department of Neurology, Vagelos College of Physicians and Surgeons, Columbia University, New York, NY, United States

**Keywords:** antimicrobial resistance, bacteria, capture sequencing, diagnostics, infectious disease

## Abstract

**Introduction:**

The “Golden Hour” is the period immediately after trauma, stroke or a cardiac event when rapid intervention is critical to reducing morbidity and mortality. The same principle should also apply to infectious diseases. Rapid, sensitive detection of infectious agents, enabling targeted interventions, has the potential to reduce mortality, morbidity, and costs of infectious diseases, and to decrease the inappropriate use of antibiotics that drives the evolution of antimicrobial resistance (AMR).

**Methods:**

Probes were designed to represent the MetaPhlAn4 database covering 894 known or potential pathogenic bacterial species, 16S rRNA sequences from the SILVA database comprising 1,325 potentially pathogenic bacterial species, genes in the Virulence Factor Database and antimicrobial resistance determinants in the Comprehensive Antibiotic Resistance Database. Target sequences were tiled with 120 nucleotide probes distributed at 60 nt intervals and clustered at 99% sequence identity. Performance measures included limits of detection (LOD) and assay reproducibility in plasma and urine using contrived and clinical samples.

**Results:**

Analytical validation using 20 bacterial strains in contrived plasma and urine samples confirmed an LOD of 5 colony-forming units per milliliter and detection of mixed infections. Results obtained with urine and blood cultures were concordant with Matrix-Assisted Laser Desorption/Ionization Time-of-Flight Mass Spectrometry (MALDI), and Blood Culture Identification (BCID) assays.

**Discussion:**

BCS2.0 enables sensitive detection of bacterial species and AMR genes and has the potential to expedite rapid, efficient infectious disease management.

## Introduction

Bacterial infections are a global health challenge. In 2019, a multicenter study reported an annual burden of 13.7 million deaths due to infections; 7.7 million were attributable to infections with 33 bacterial pathogens (GBD 2019 Antimicrobial Resistance Collaborators, 2022). Antimicrobial resistance (AMR) infections are an important factor in morbidity and mortality (Antimicrobial Resistance Collaborators, 2022). In the United States alone, the annual burden of AMR is estimated at 2.8 million infections, 35,000 deaths, with a direct cost of $4.6 billion and $55 billion in lost productivity (Dadgostar, 2019; Nelson et al., 2021; Flynn and Guarner, 2023). Culture-based and phenotypic antimicrobial susceptibility testing remain the gold standard for diagnosis. These assays are resource-intensive and require 1–3 days to deliver the insights needed to direct the efficient use of antibiotics. Multiplex polymerase chain reaction (PCR) panels have been established for bacterial identification and detection of the AMR genes in a time frame of less than 1 hour. In concert, these panels can identify up to 26 bacterial agents and a maximum of 19 antimicrobial resistance targets (Ozongwu et al., 2017; Devrim et al., 2025).

Untargeted metagenomic sequencing (untargeted high-throughput sequencing, UHTS) can detect all bacteria present in a sample. However, UHTS requires shipping to centralized laboratories with dedicated staff and substantial computational power and expertise for data analysis. Like traditional culture methods, the time frame from sample collection to delivery of results is 1–3 days.

Targeted-capture sequencing is an alternative strategy for pathogen identification that bridges performance gaps in breadth of analysis, cost, complexity and time required between multiplex PCR and metagenomic sequencing (Kim et al., 2025; Yi et al., 2025). We have reported its utility in diagnosis of viral infections and obtained regulatory approval for use of this platform (Viral Capture Sequencing, VCS) in New York State (Briese et al., 2015; Kapoor et al., 2024). In BacCapSeq (BCS), we extended capture sequencing to target the proteomes, virulence factors, and AMR factors in 307 bacterial species (Allicock et al., 2018). We used the same whole proteome tiling strategy to develop the first BCS probe set that we used in building VCS. Setting a limit of 4 M probes, we could target only 307 bacteria. Our ultimate goal is to build a single pan-microbial system that can detect bacteria, viruses, fungi and parasites. We recently reduced the VCS system to less than 1 million probes without loss of functionality through modifications in probe length and target selection (Kapoor et al., 2024). We used a distinct approach in BCS2.0 by targeting only specific regions essential for species definition and clinically important regions (AMR and virulence genes). The result was a reduction in probe numbers from 4.2 million in BCS to <1 million in BCS2.0. Here, we describe the development and validation of an enhanced version of BCS that further increases efficiency, reduces redundancy, and minimizes complexity in data analysis.

## Materials and methods

### Bacterial species

We selected 20 clinically important bacterial strains, representing 15 species, to validate bacterial detection using BCS2.0. These included *H. influenzae, M. catarrhalis, L. pneumophila*, *N. meningitidis, S. agalactiae, S. pyogenes, L. monocytogenes*, *E. coli, K. pneumoniae, P. mirabilis, E. faecalis*, *S. aureus, P. aeruginosa, A. baumannii* and *M. intracellulare* (Table 1).

**Table 1:** Details of bacterial strains used in validation.

S. No	Bacterial species	Strain designation	Gram strain
1.	*Acinetobacter baumannii*		−ve
2.	*Enterococcus faecalis*	NJ-3	+ve
3.	*Escherichia coli*	FDA Seattle 1946	−ve
4.	*Escherichia coli*	NDM-1	−ve
5.	*Escherichia coli*	J53 pMG224	−ve
6.	*Hemophilus influenzae type B*	AMC 36-A-1	−ve
7.	*Klebsiella pneumoniae*	NCTC 9633	−ve
8.	*Klebsiella pneumoniae*	ART 2008133	−ve
9.	*Klebsiella pneumoniae*	bMx# 1103199	−ve
10.	*Klebsiella pneumoniae*	1000527, 7561	−ve
11.	*Legionella pneumophila*	Philadelphia-1	−ve
12.	*Listeria monocytogenes*	53 XXIII	+ve
13.	*Moraxella catarrhalis*	NCTC 11020	−ve
14.	*Mycobacterium intracellulare*	TMC 1406	+ve (AFB)*
15.	*Neisseria meningitidis serogroup A*	NCTC 10025	−ve
16.	*Pseudomonas aeruginosa*	NCTC 10332	−ve
17.	*Proteus mirabilis*	LRA 08 01 73	−ve
18.	*Staphylococcus aureus*	NCTC 8532	+ve
19.	*Streptococcus agalactiae*	NCTC 8181	+ve
20.	*Streptococcus pyogenes*	CIP 104226	+ve

*AFB-acid fast bacilli.

*Probe design*: we designed BCS2.0 for the efficient, sensitive, and cost-effective sequence-based detection of pathogenic bacteria and associated antimicrobial resistance (AMR) and virulence factors. Our design incorporated four major components: (1) Clade-specific marker genes: unique marker gene sequences from the MetaPhlAn4 database (vOct2022) (Blanco-Miguez et al., 2023), covering 894 known or potential pathogenic bacterial species (Supplementary Table S1); (2) 16S rRNA genes: representative 16S rRNA sequences from the SILVA database (138.1) (Quast et al., 2013), encompassing 1,325 potentially pathogenic bacterial species; (3) Virulence factors: all known virulence factor genes curated in the Virulence Factor Database (VFDB, December 2022) (Liu et al., 2022); (4) Antimicrobial resistance genes: all known antimicrobial resistance determinants from the Comprehensive Antibiotic Resistance Database (CARD, v3.2.5) (Supplementary Table S1) (McArthur et al., 2013; Alcock et al., 2020). The sequences of interest were extracted from each dataset using customized bash and R scripts. The combined target sequences comprised 166,353 genetic fragments, including 155,944 unique marker sequences from 894 bacterial species recorded in Metaphlan4, 1,325 from SILVA 16S, 4,750 from CARD, and 4,334 virulence-related sequences from VFDB. These target sequences were then tiled with 120 nucleotide probes distributed at 60 nt intervals and clustered at 99% sequence identity with mmseqs or CD-HIT.

### Sample preparation and nucleic acid extraction

Contrived samples were prepared by spiking bacteria into a negative sample matrix (pooled human urine or plasma). Plasma was obtained from the New York Blood Center from five individual donors. Pooled human urine was commercially sourced (Innovative Research). The contrived samples consisted of a series of bacterial dilutions ranging from 5 × 10^4^ CFU/mL to 5 CFU/mL in a negative sample matrix. Plasma or urine without bacteria served as negative experimental controls. Lysis was performed by incubating the samples with easyMAG lysis buffer for 10 min at room temperature. Total nucleic acid (TNA) was extracted using the bioMérieux easyMAG system. Nucleic acid quantity and quality were assessed using a NanoDrop (ThermoFisher) and a Qubit (ThermoFisher).

### Library preparation, enrichment and sequencing

TNA was reverse transcribed to cDNA using Superscript IV (ThermoFisher) and Klenow fragment (NEB). cDNA was quantified using Qubit prior to enzymatic fragmentation. Fragments were ligated with adapters and indexed using barcoded primers to generate 350–450 bp libraries. Libraries were quantified using Tapestation (Agilent), and 8–10 samples containing 200 ng each sample were pooled. The pooled libraries were incubated with biotinylated BCS2.0 probes (Twist Biosciences) for an hour at 60 °C. Following incubation with streptavidin-coated beads, the enriched pools were collected using a magnet. After elution, the libraries were amplified by polymerase chain reaction and sequenced on the Illumina NextSeq 2000 system to generate 150 bp single-end reads. A detailed protocol is described in our previous study (Briese et al., 2015; Kapoor et al., 2024).

### Bioinformatic analysis

*Bacterial identification*: raw reads were demultiplexed and adapters trimmed using Cutadapt (Martin, 2011). The reads were then quality-filtered and end-trimmed using PRINSEQ (Schmieder and Edwards, 2011). Residual host reads that survived the capture enrichment were removed through mapping of filtered reads to the human reference database using Bowtie2 (Langmead and Salzberg, 2012). The *de novo* assembly of host-subtracted reads was performed using MEGAHIT (Li et al., 2015), and all contigs and singletons were subjected to a homology search against the GenBank nucleotide (nt) database using MEGABLAST (Morgulis et al., 2008) and mapped to a custom database of our targeted marker sequences. Three negative experimental controls were included in each sequencing run to account for potential contamination from experimental or reagent sources. We reformatted results from MegaBlast and custom mapping to input into the contamination-detection R package known as “decontam” (Davis et al., 2018). We used prevalence-based detection of contamination at the threshold *p* = 0.5 within decontam. For robust analysis, we included all species with at least 20 reads and 100 iterations were run, with random subsampling of samples and negative experimental controls at 60% in each iteration. Species present in 80 of 100 iterations were eliminated as contaminants. The assignment of a taxonomic designation required a minimum of 20 reads that mapped to at least 5% of the core-genomic marker regions. In instances where mapping results indicated more than one species within the same genus, we used Megablast to eliminate ambiguity. Thus, final taxonomic affiliation was determined based on comparisons of mapping and Megablast results (Morgulis et al., 2008; Blanco-Miguez et al., 2023).

### Genetic antimicrobial resistance and virulence determination

The presence of virulence factors was investigated by aligning the assembled contigs to the VFDB databases using blastn. For AMR analyses, two stringent approaches were used. In the first approach, the contigs generated were run through the Resistance Gene Identifier (RGI) (McArthur et al., 2013) using default settings to generate a results table. No loose hits were included in order to exclude partial and low coverage AMR genes. Genes passing the following three criteria were marked as present: (i) perfect hits in any model; (ii) protein homologue model with >80% identity and a ratio >1 for best hit bit score/passing bitscore; (iii) In protein variant model, identity >85% and have a known SNP. In the second approach, all host-subtracted reads were mapped to the RGI database using k-mer alignment (KMA) (Clausen et al., 2018), and genes with an identity of at least 85%, a coverage of at least 80%, and a depth of one were marked as present. When multiple alleles of the same gene were present, only the best hit was reported. Final AMR results were reported as tables showing the genes associated with the identified antimicrobial class for each sample.

### Analytical validation strategy

The validation strategy was adapted from the New York State Department of Health (NYSDOH) and Clinical Laboratory Improvement Amendments (CLIA) guidelines. This consisted of the following steps:

1) *LOD determination*: the LOD was determined using the contrived samples in two matrices: plasma and urine. For each matrix, bacteria quantified as colony-forming units were serially diluted from 5 × 10^4^–5 CFU/mL and processed with BCS2.0 sample preparation, then sequenced as described above.
2) *Assay precision*: to determine the precision of BCS2.0, we assessed repeatability and reproducibility. Separate samples were processed, and the assay was run in triplicate by three different operators at three different experiment dates, at 5X the determined LoDs for each bacterium in both matrices.
3) *Accuracy*: to determine assay accuracy, we used contrived urine and plasma samples. Two bacterial species were mixed at two concentrations (20 CFU and 1,000 CFU) and prepared as described previously. In some instances, we also mixed three different bacteria at equal concentrations (100 CFU) and performed an assay. The background was detected using the bioinformatic tool decontam, and results were shown as specific bacterial reads per million raw reads for each of the mixed bacteria.

### Clinical concordance

Clinical samples were obtained from the clinical microbiology service lab at New York-Presbyterian/Columbia University Irving Medical Center, New York City. The samples comprised 56 primary urine samples and 31 blood culture samples from bottles. In instances where clinical plasma samples containing specific bacteria were not available, we used contrived samples at the LOD to evaluate assay performance as instructed in the microbiology molecular checklist provided by the New York State Department of Health, Wadsworth Center^1^. The laboratory test results for urine samples were based on isolated colony identification from bacterial culture plates, followed by MALDI-based or biochemical species identification and antibiotic susceptibility testing (AST) in microtiter plates (MicroScan, Beckman Coulter) for minimal inhibitory concentration (MIC) determination were based on CLSI breakpoints. For blood culture, the results included MALDI-based species determination or biochemical identification from isolated colonies on bacterial culture plates as well as multiplex PCR results directly from positive blood culture bottles (BCID, Blood Culture Identification panel, Biomerieux).

## Results

### In silico analysis of probe design

The BCS2.0 system comprises a total of 988,786,120-mer nucleotide probes across 166,353 genetic elements (loci). These regions cover a total of 1,335 clinically relevant and potentially pathogenic bacterial species either through clade-specific marker sequences or full-length 16S rRNA sequences (Supplementary Figure S1). We also evaluated the uniqueness of probes between two species within the same genus, *S. aureus* and *S. hominis*, and found that less than 10% of probes had overlap. The in-silico hybridization efficiency was also evaluated by blast analysis of probes to 50 randomly selected genome sequences of *S. aureus* and *S. hominis* isolates. This showed that an average of 85% of probes for each specific bacterium mapped to these 50 genomes (Supplementary Figure S1).

### Sensitivity for the detection of bacteria

**Figure 1:**
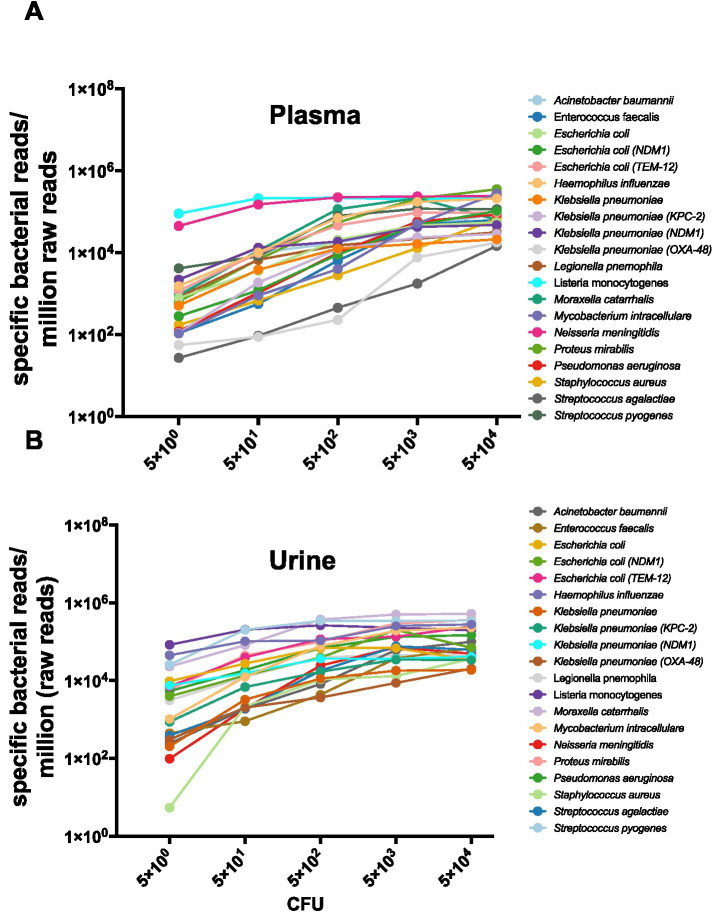
Linearity data for 20 bacterial strains in contrived plasma **(A)** and urine **(B)**. Bacterial strains were serially diluted in matrix (plasma or urine), and specific bacterial reads mapping only to targeted bacteria were shown as specific bacterial reads per million raw reads.

We first compared the efficiency of BCS2.0 and UHTS using contrived plasma samples spiked with *E. coli, S. aureus and S. agalactiae*. For all three species evaluated (*E. coli, S. aureus and S. agalactiae*), BCS2.0 consistently yielded higher read counts than UHTS. The enhancement in sensitivity was most pronounced at lower bacterial concentrations (>1,500 fold) (Supplementary Table S2). Whereas at 1 × 10^3^ CFU/mL of these three bacteria, UHTS detected <2,300 genomic reads/M raw reads and <150 targeted bacterial reads/M raw reads, BCS2.0 detected >40,000 genomic reads/M raw reads and >25,000 targeted bacterial reads/M raw reads. This corresponds to an increase of minimum 95-fold for genomic reads and 720-fold for targeted bacterial reads across these three species (Supplementary Table S2). We then determined the performance of BCS2.0 for the detection of 20 bacterial species in plasma and urine samples using bacterial cultures (CFU/ml) diluted serially 10-fold over a range of 5 × 10^4^ CFU/mL to 5 CFU/mL. All 20 bacterial species were detected at a LoD of 5 CFU/mL (Supplementary Table S3). Figure 1 shows the linearity of data obtained for plasma (Figure 1A) and urine (Figure 1B).

### Reproducibility of assay performance

**Figure 2:**
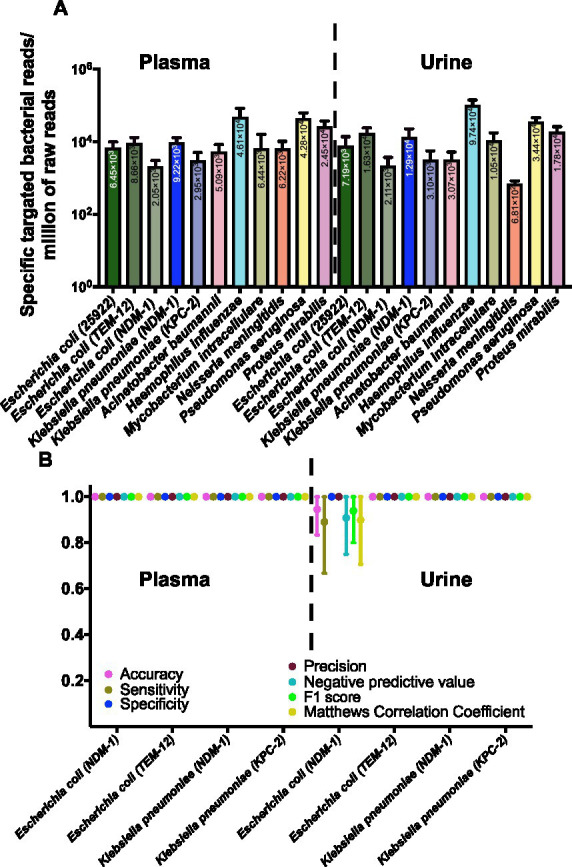
Assessment of BCS2.0 assay performance. Repeatability and reproducibility for the detection of bacteria **(A)** were done on 11 representative bacteria in plasma (left) and urine (right). Antimicrobial resistance performance **(B)** was assessed for four bacterial strains carrying *bla* genes (*bla*_NDM-*1*_*, bla*_KPC-2_, and *bla*_TEM-12_ in contrived plasma (left) and urine (right). All data for bacterial detection were from three technical replicates performed by three different operators on different dates and are presented as mean specific bacterial reads per million raw reads. Error bars represent the standard deviation between the replicates. The metrics for AMR gene detection are presented after bootstrapping (1000) on initial triplicate experiments.

To evaluate assay reproducibility, we conducted precision testing using three replicates of 11 representative bacterial species. The assay was performed in triplicate by three different operators on three different dates at 5X LoD. This panel consisted of eight species, including four resistance strains that carry the beta-lactamase (*bla*) *bla*TEM-12, *bla*NDM-1, and *bla*KPC-2 genes. At 5X LOD, all inter-run and intra-run samples exceeded the 5% minimum coverage required for bacterial species determination in both contrived plasma and urine samples (Supplementary Table S4). Figure 2A represents the performance of BCS2.0 at 5X LoD for 11 targets in plasma and urine. In plasma, the median coefficient of variance (CV) was 8.8%, with a standard deviation (SD) of 0.33 (log10). Although there was variability across three biological replicates for *M. intracellulare* and *N. meningitidis* in plasma, both species were consistently detected within the defined criteria for species designation in the BCS2.0 assay and results were concordant across three biological replicates. In urine, the median coefficient of variation (CV) was 10.4%; the median standard deviation (SD) was 0.35 (log10). All bacteria showed <25% CV, indicating consistent assay precision (Supplementary Figure S2). The four bacterial strains harboring *bla*NDM-1 and *bla*TEM-12 in *E. coli* and *bla*KPC2 and *bla*NDM-1 in *K. pneumoniae* were used to determine sensitivity and specificity using contrived specimens with known positive and negative results (Supplementary Figure S2). The results showed that, for four bacterial species in both matrices, the assay revealed 100% specificity and precision. For *E. coli* with *bla*NDM-1 in urine, there was one instance in which *bla*NDM-1 was not detected with high confidence, due to stringent criteria for gene identification (Supplementary Table S4). For robust analysis, we estimated assay performance uncertainty using stratified bootstrap resampling (1000). Positive and negative samples were resampled within each bootstrap to preserve the original distribution. Mean performance metrics are reported in Figure 2B. Accuracy, sensitivity, negative predictive value, F1 score and Mathew’s correlation coefficient were 100% for all strains, except for *E. coli* (*bla*NDM-1) in urine, where it was 0.94, 0.89, 0.90, 0.93, and 0.89 (Figure 2B). Overall, these performance metrics provide robust and consistent coverage of targeted bacterial reads for reliable qualitative detection of bacteria and AMR genes in plasma and urine.

### Detection of mixed infections

We evaluated the performance of BCS2.0 for detecting two or more bacterial species in single samples. We prepared a total of 41 contrived mixed-infection samples in plasma (*n* = 18) and urine (*n* = 23). In samples representing two different bacterial species, we included one species at a concentration at 20 CFU/mL (low) and the other at 1000 CFU/mL (high). In samples representing three different bacterial species, each species was introduced at a concentration of 100 CFU/mL. In all mixed samples in both matrices, we detected all spiked bacterial species (Tables 2, 3).

**Table 2:** Detections of co-infections in plasma mixes of two or more bacteria.

Mix	Bacteria	CFU/ml	Raw reads	Target reads/M raw reads	% Target coverage	BCS2.0 call
M1	*Pseudomonas aeruginosa*	1,000	8,854,799	89,482	97.9	+
	*Klebsiella pneumoniae (KPC-2)*	20	8,854,799	252	92.83	+
M2	*Mycobacterium intracellulare*	1,000	8,343,357	74,443	89.77	+
	*Escherichia coli (NDM-1)*	20	8,343,357	49	10.04	+
M3	*Escherichia coli (TEM-12)*	1,000	27,982,935	53,383	93.45	+
	*Acinetobacter baumannii*	20	27,982,935	68	28.17	+
M4	*Escherichia coli (TEM-12)*	1,000	9,423,870	2,186	80.01	+
	*Acinetobacter baumannii*	20	9,423,870	6,864	64.94	+
M5	*Escherichia coli (NDM-1)*	1,000	11,634,007	32,012	87.94	+
	*Mycobacterium intracellulare*	20	11,634,007	1713	84.23	+
M6	*Escherichia coli (TEM-12)*	1,000	22,992,542	50,061	95.39	+
	*Neisseria meningitidis*	20	22,992,542	299	75.07	+
M7	*Mycobacterium intracellulare*	1,000	9,978,584	117,850	89.2	+
	*Haemophilus influenzae*	20	9,978,584	33,963	93.23	+
M8	*Haemophilus influenzae*	1,000	34,181,313	276,757	96.44	+
	*Mycobacterium intracellulare*	20	34,181,313	363	83.48	+
M9	*Haemophilus influenzae*	1,000	5,337,342	37,437	92.55	+
	*Neisseria meningitidis*	20	5,337,342	64,744	94	+
M10	*Haemophilus influenzae*	1,000	20,314,290	232,483	96.13	+
	*Escherichia coli (NDM-1)*	20	20,314,290	112	42.78	+
M11	*Neisseria meningitidis*	1,000	3,532,400	32,716	90.93	+
	*Escherichia coli (TEM-12)*	20	3,532,400	1775	63.86	+
M12	*Escherichia coli (NDM-1)*	20	52,733,099	143	61.38	+
	*Haemophilus influenzae*	1,000	52,733,099	303,034	96.45	+
M13	*Neisseria meningitidis*	1,000	6,554,671	99,354	94.22	+
	*Mycobacterium intracellulare*	20	6,554,671	3,616	84.05	+
M14	*Haemophilus influenzae*	100	13,276,216	40,208	91.08	+
	*Neisseria meningitidis*	100	13,276,216	2,117	84.48	+
	*Klebsiella pneumoniae (KPC-2)*	100	13,276,216	3,179	97.25	+
M15	*Streptococcus pyogenes*	100	32,207,279	52,726	94.79	+
	*Neisseria meningitidis*	100	32,207,279	847	84.83	+
	*Klebsiella pneumoniae (NDM-1)*	100	32,207,279	12,235	97.61	+
M16	*Klebsiella pneumoniae (KPC-2)*	100	12,170,305	7,287	97.37	+
	*Acinetobacter baumannii*	100	12,170,305	5,967	66.36	+
	*Proteus mirabilis*	100	12,170,305	17,167	88.23	+
M17	*Haemophilus influenzae*	100	15,433,265	102,756	94.86	+
	*Pseudomonas aeruginosa*	100	15,433,265	51,351	97.85	+
	*Neisseria meningitidis*	100	15,433,265	5,093	87.82	+
M18	*Escherichia coli (NDM-1)*	100	6,272,260	4,614	76.99	+
	*Acinetobacter baumannii*	100	6,272,260	20,417	70.89	+
	*Proteus mirabilis*	100	6,272,260	42,298	89.99	+

The first column indicates mixtures (M) of individual bacterial species.

**Table 3:** Detections of co-infections in urine mixes of two or more bacteria.

Mix	Bacteria	CFU/ml	Raw reads	Target reads/M raw reads	% Target coverage	BCS2.0 call
M1	*Escherichia coli (TEM-12)*	1,000	8,196,752	177,031	94.18	+
	*Acinetobacter baumannii*	20	8,196,752	334	40.66	+
M2	*Acinetobacter baumannii*	1,000	6,704,498	55,170	87.14	+
	*Escherichia coli (TEM-12)*	20	6,704,498	10,316	87.82	+
M3	*Escherichia coli (NDM-1)*	1,000	33,464,078	63,193	92.6	+
	*Acinetobacter baumannii*	20	33,464,078	1,496	63.23	+
M4	*Acinetobacter baumannii*	1,000	8,887,689	57,481	87.69	+
	*Escherichia coli (NDM-1)*	20	8,887,689	1,083	69.21	+
M5	*Klebsiella pneumoniae (NDM-1)*	1,000	25,931,696	45,330	97.88	+
	*Escherichia coli (25922)*	20	25,931,696	724	51.09	+
M6	*Escherichia coli (25922)*	1,000	26,109,651	45,213	61.46	+
	*Klebsiella pneumoniae (NDM-1)*	20	26,109,651	650	96.06	+
M7	*Proteus mirabilis*	1,000	12,490,344	287,321	97.75	+
	*Klebsiella pneumoniae (KPC-2)*	20	12,490,344	1,579	96.74	+
M8	*Klebsiella pneumoniae (KPC-2)*	1,000	26,110,544	35,214	97.83	+
	*Proteus mirabilis*	20	26,110,544	2,944	84.66	+
M9	*Klebsiella pneumoniae (KPC-2)*	1,000	11,595,108	15,987	97.79	+
	*Proteus mirabilis*	20	11,595,108	2,806	87.99	+
M10	*Proteus mirabilis*	1,000	16,570,426	237,941	98.04	+
	*Klebsiella pneumoniae (NDM-1)*	20	16,570,426	8,135	97.77	+
M11	*Klebsiella pneumoniae (NDM-1)*	1,000	32,529,495	27,023	97.87	+
	*Acinetobacter baumannii*	20	32,529,495	67	47.98	+
M12	*Acinetobacter baumannii*	1,000	11,352,866	102,557	94	+
	*Klebsiella pneumoniae (KPC-2)*	20	11,352,866	9	19.43	+
M13	*Klebsiella pneumoniae (KPC-2)*	1,000	16,207,338	20,796	97.84	+
	*Acinetobacter baumannii*	20	16,207,338	646	68.85	+
M14	*Acinetobacter baumannii*	1,000	15,913,015	95,513	96.26	+
	*Proteus mirabilis*	20	15,913,015	8,368	95.67	+
M15	*Proteus mirabilis*	1,000	13,730,183	334,565	97.99	+
	*Acinetobacter baumannii*	20	13,730,183	1,695	69.94	+
M16	*Pseudomonas aeruginosa*	1,000	33,717,432	132,727	97.99	+
	*Acinetobacter baumannii*	20	33,717,432	512	67.96	+
M17	*Pseudomonas aeruginosa*	1,000	30,353,903	113,769	97.97	+
	*Klebsiella pneumoniae (KPC-2)*	20	30,353,903	176	95.9	+
M18	*Klebsiella pneumoniae (NDM-1)*	1,000	27,317,169	27,073	97.86	+
	*Pseudomonas aeruginosa*	20	27,317,169	410	90.94	+
M19	*Proteus mirabilis*	100	7,177,089	51,694	94.95	+
	*Acinetobacter baumannii*	100	7,177,089	33,144	90.01	+
	*Klebsiella pneumoniae (KPC-2)*	100	7,177,089	13,827	97.45	+
M20	*Pseudomonas aeruginosa*	100	11,897,946	102,221	97.93	+
	*Escherichia coli (TEM-12)*	100	11,897,946	22,096	92.06	+
	*Mycobacterium intracellulare*	100	11,897,946	16,354	88.37	+
M21	*Moraxella catarrhalis*	100	16,763,715	19,530	95.35	+
	*Klebsiella pneumoniae (NDM-1)*	100	16,763,715	31,973	97.88	+
	*Haemophilus influenzae*	100	16,763,715	51,686	95.49	+
M22	*Escherichia coli (25922)*	100	10,642,142	38,350	75.83	+
	*Proteus mirabilis*	100	10,642,142	20,342	92.45	+
	*Acinetobacter baumannii*	100	10,642,142	4,160	66.4	+
M23	*Neisseria meningitidis*	100	15,646,258	1,069	84.14	+
	*Escherichia coli (25922)*	100	15,646,258	34,025	65.99	+
	*Streptococcus pyogenes*	100	15,646,258	92,548	96.89	+

The first column indicates mixtures (M) of individual bacterial species.

### Concordance with clinical microbiology results in blinded analyses

We compared results obtained with BCS2.0 and current standard of care (SOC) microbiology results obtained in the clinical microbiology laboratory at the Columbia University Irving Medical Center (CUIMC).

**Figure 3:**
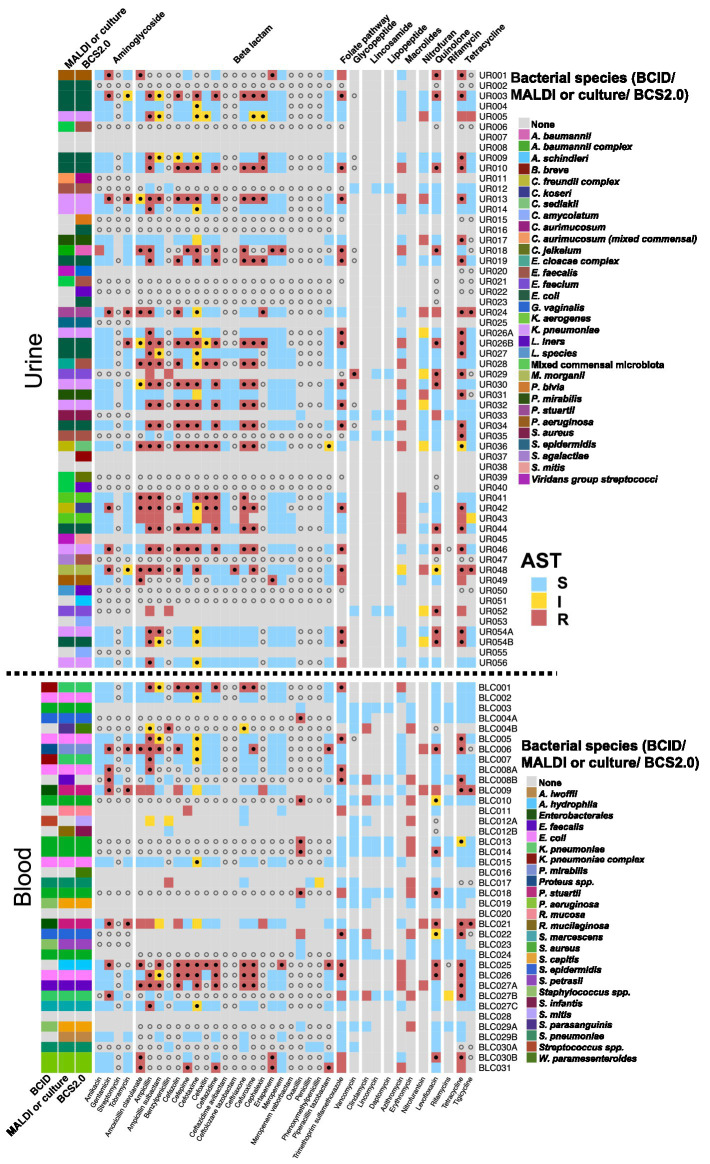
Clinical concordance for bacterial detection and antimicrobial resistance in urine (top) and blood cultures (bottom). For urine samples, clinical results included identification by MALDI and an AST profile for the antibiotics listed at the bottom. Antimicrobial classes are shown at the top of the panel. For blood cultures, BCID results were also presented alongside MALDI and BCS2.0 results. Blue squares represent sensitive; yellow squares represent intermediate resistance; red squares represent resistance; and grey squares indicate that the antibiotic was not tested. Solid dots represent AMR genes associated with that group in either resistant or intermediate-resistant strains. For grey squares, hollow circles represent a probable AMR gene detected by the BCS2.0 assay in that class. When a sensitive phenotype was present, no AMR genes were overlaid on it. BCS2.0 reported bacteria only if they were detected in either a phenotypic assay, and the complete list of bacteria was presented in Supplementary Table S5. Each bacterium is represented by a different color, as indicated by the color legends adjacent to respective panels.

We examined 56 urine culture samples; 45 culture-positive samples and 11 culture-negative samples. Matrix-Assisted Laser Desorption/Ionization Time-of-Flight Mass Spectometry (MALDI) and/or biochemical testing was used for definitive bacterial identification (ID). AST was performed by microdilution. Mixed commensal bacteria were reported if there were more than three bacterial species present. All bacterial species that were marked as contaminants in each run were removed from analyses (Supplementary Table S5), except when a species in the sample mapped more than 5-fold unique reads than in negative samples. In all 45 culture-positive cases, BCS2.0 found the same bacteria as reported in SOC results. Moreover, in cases where mixed commensal bacteria were reported, BCS2.0 identified the same bacterial profile that would be reported as mixed commensal bacteria. For example, whereas UR006 had *E. faecalis*, *E. coli*, *E. gallinarum*, *E. avium*, and *S. thermophilus* as predominant organisms in BCS2.0 analysis, SOC was reported as “mixed commensal microbiota”, showing that BCS2.0 can also use the same metrics used by SOC to identify mixed bacterial cultures. The genotypic AMR profile included fluoroquinolone (*emrB, CRP, mdtE*) (Lomovskaya and Lewis, 1992; Nishino et al., 2008; Soto, 2013), tetracycline (Tet) (Roberts, 2005), beta-lactam antibiotic (*marA, AcrE, Tem-1*) (Cohen et al., 1988; Lau and Zgurskaya, 2005; Salverda et al., 2010), aminoglycoside [*APH(3″*)-I′, APH(3′)-IIIa APH(6)-Id] (Trieu-Cuot and Courvalin, 1983; Fong and Berghuis, 2009), and macrolide resistance (*mphA*) genes (Supplementary Table S5). BCS2.0 was more precise than MALDI. In select cases where MALDI obtained only group-level designations, BCS2.0 provided species-level identification (e.g., UR028: *E. cloacae* complex – *E. cloacae, E. mori, E. kobei*) (Supplementary Table S5). We detected one or more genetic determinants for almost all classes of antibiotics phenotypically assayed (Figure 3). An example is a sample containing vancomycin-resistant *E. faecium* (UR029) identified in AST, that was found in BCS2.0 to harbor VanR, VanH, VanY, and VanZ genes encoding vancomycin resistance (Supplementary Table S5) (Courvalin, 2006). In another instance of ertapenem and meropenem-resistant *A. baumanii* (UR018), BCS2.0 identified OXA-23 as carbapenem resistance gene (Donald et al., 2000). Additionally, *adeL*, *adeA*, *adeB*, *adeF*, *adeG*, *adeH*, *adeI*, *adeJ*, *adeK*, and *PDC-5* were present, which reflect a multidrug resistance genotype (Supplementary Table S5) (Rodriguez-Martinez et al., 2009; Lari et al., 2018). In summary, BCS2.0 was in agreement with ID and AST in urine analyses but provided higher-resolution insights into species-level identification, mixed-infection, and antibiotic-resistance profiles.

We examined a total of 31 blood culture samples: 28 culture-positive samples and three culture-negative samples (Supplementary Table S5). In blood culture samples, the routine lab tests that were conducted were the following: BioFire Blood Culture Identification (BCID) direct from the positive blood culture bottle, MALDI and/or biochemical testing for bacterial ID from isolated colonies 24 h after the BCID results, and AST for detection of antibiotic resistance following ID from isolated colonies. The samples included one-, two-, and multi-species infections. Twenty-two pathogens were identified by BCID and ID. We observed seven instances wherein BCID and ID differed. In each of these instances, BCS2.0 detected the bacterial agent identified in either BCID or ID. For example, whereas BCID did not report any bacteria in sample BLC011, *R. mucosa,* an agent not represented in the BCID panel, was detected by ID. BCS2.0 identified not only *R. mucosa* but also *R. gliardii*. Similarly, in BLC029, *A. lwoffii* and *S. capitis* were detected by ID from isolated colonies, and BCID identified *Staphylococcus* spp., whereas BCS2.0 identified both *S. capitis* and *A. lwoffii*. In one blood culture (BLC027), three *bacteria* (*E. faecalis, K. pneumoniae and S. marcescens*) were identified by BCID and both ID and BCS2.0 confirmed the presence of all three isolates. Furthermore, blood culture samples yielded gene determinants for all phenotypic antibiotics for which resistance was found (Figure 3 and Supplementary Table S5).

## Discussion

Antibiotics are the mainstay in clinical management of bacterial infections. Accordingly, improvements in diagnostic methods for rapidly implicating specific agents and detecting their AMR profiles have the potential to reduce morbidity, mortality, and health care costs, as well as the inappropriate use of antibiotics that drives the emergence of AMR (Podolsky, 2018; Kaprou et al., 2021).

Advancements in molecular methods have changed the landscape of infectious agent detection, but culture-based methods remain the gold standard (Burnham et al., 2017; Yamin et al., 2023). The most widely used molecular assays in clinical microbiology after culture-based methods are PCR-based assays (Yamin et al., 2023). PCR assays can be sensitive and quantitative, but they have limitations (Anjum et al., 2017). They can only detect a limited number of targets. Additionally, because they rely on specific binding events, PCR assays are sensitive to sequence variation in primer (and probe) binding sites (Salimnia et al., 2016; Timmermans et al., 2022; Shin et al., 2023). Finally, PCR assays do not provide sequence information. UHTS sequencing has no phylogenetic constraints (Ferreira et al., 2023) but is resource-intensive and less sensitive than PCR (Chen et al., 2022).

Capture sequencing retains the agnostic advantages of UHTS without sacrificing sensitivity (Sanchez-Vicente et al., 2022; Kapoor et al., 2024). Because capture sequencing is focused, it is up to 1,000X more sensitive (5 copies/ml) than UHTS (5,000 copies/ml). It facilitates the detection of pathogens at low levels in clinical and environmental samples that may be missed with UHTS. The BCS panel described here can identify over 1,300 bacterial species and their associated AMR genes with high precision. BCS2.0 received CLEP certification from NYSDOH and is available for clinical uses.

We have reported here the validation of BCS2.0 in urine and plasma. Except for one instance in urine, we recovered AMR genes (*bla*NDM-1, *bla*TEM-12, and *bla*KPC-2) from four strains of *E. coli* and *K. pneumoniae* in both matrices. We use a stringent criterion for high-confidence AMR gene detection, and occasional misses were observed when the *bla*NDM-1 cutoff was not met. We speculate that non-specific enzymatic fragmentation during library preparation may contribute to failure due to the loss of genetic sequence continuity. In 3 cases, BCS2.0 detected a full-length *mecA* gene; in 1 instance, *mecA* was identified at 43.9% identity and 45.79% coverage but did not meet BCS2.0 criteria for a positive call. In two instances, we found *bla*CTX-M in blood; *bla*CTX-M15 was confirmed in both samples by BCS2.0. Christians et al. reported positive percent agreement and diagnostic yield of 83.3 and 71.4% for *bla*CTX-M detections (Christians et al., 2024). Our results indicate similar assay performance and provide ease of analysis.

In our blood culture testing, we observed discrepancies between BCID and MALDI-based identification systems in the clinical laboratory. In each case, BCS2.0 identified pathogens at the species level and genotypic markers of AMR. Although BCS2.0 uses a stringent criterion for high-confidence detection of AMR genes, it can be flexibly customized to adapt and take calls by lowering thresholds, providing users with flexibility in analysis and interpretation when sequencing depth is lower. Moreover, BCS2.0 provided species designations when group-level identifications or no identifications were made by MALDI or BCID (e.g., *P. stuartii, P. mirabilis, S. mitis*) (Figure 3 and Supplementary Table S5).

Genotypic and phenotypic analyses of AMR can be discordant (Yee et al., 2021; Rebelo et al., 2022). The presence of a gene does not always result in a phenotype, and a phenotype may not always be linked to the same genotype (Hughes and Andersson, 2017; Livermore et al., 2020; Chetri, 2023). Accordingly, the presence or absence of an AMR gene should not be the sole guide for antibiotic therapy. In our clinical samples, we observed two instances of carbapenem resistance (UR018 and BLC025). In both cases, we detected the presence of the carbapenemase genes OXA-23-like and KPC-3 (Woodford et al., 2004). Whereas MALDI identified only *A. hydrophila*, BCS2.0 detected additional *Aeromonas* species.

Culture and BCS results were largely concordant. We identified all bacteria reported in urine and blood culture reports; however, BCS also detected bacteria not detected by culture. In one instance in which blood culture reports were negative, BCS2.0 identified *W. paramesenteroides*. BCS2.0 also identified an additional four cases of *W. paramesenteroides* in blood cultures. We speculate that failure to detect *Weissella* species is due to their fastidious nutritional and temperature requirements for growth (Abriouel et al., 2015; Fusco et al., 2015). At present, implementation of BCS2.0 requires shipment of samples to a CLIA certified laboratory. We are developing a fully integrated automated system that extracts and sequences clinical and environmental samples. However, this system is not yet available for clinical use. The cost for extraction, library preparation, probes and sequencing kits of a single sample is approximately $200 US. Costs for running multiple samples simultaneously are lower. All software used are freely available and are version controlled to meet clinical requirements.

Another critical challenge with culture- and UHTS-based detection systems is the time required to process and deliver results. Following a blood culture bottle becoming positive, the current time for culture-based methods is 48 h or more from the time a pathogen is initially identified to when the AST/AMR results are finally available. In a recent collaboration with the Illumina Solutions Center in Baltimore, we completed a VCS run from sample extraction to delivery of sequence data using a MiSeq i100 in less than 14 hours.

The VCS and BCS probe sets have been combined into a single system comprising <2 M probes that does not sacrifice sensitivity for detection of either viral or bacterial targets (data not shown). An extension to fungi is underway. Integration of these elements into a pan microbial platform will enable rapid, inexpensive differential diagnosis of infectious diseases.

## Data Availability

The original contributions presented in the study are publicly available. This data can be found here: https://www.ncbi.nlm.nih.gov/bioproject/PRJNA1448054/.

## Footnotes

1
https://www.wadsworth.org/sites/default/files/WebDoc/Microbiology_NAAT_Checklist_0211.pdf
