# Supplementary material for: BCS2.0: a capture sequencing platform for rapid differential diagnosis of bacterial infections and antimicrobial resistance
Source: Front Microbiol. 2026 Apr 20;17:1802485. doi: 10.3389/fmicb.2026.1802485 (PMC13136086; doi:10.3389/fmicb.2026.1802485)
Supplement: Supplementary file 1 [file Image_1.PDF]

Figure S1

Insilco validation of probe design. Overlapping marker sequences between the species of same genus *Helicobacter* (A) and *Staphylococcus*. *H magdebungensis* and *H. cinaedi* belong to a same group and are identified by same sequences. Numbers of overlapping probes for *S. aureus* and *S. hominis*. (D) Mapping of species-specific probes to 50 genomes of *S. aureus* (left) and *S. hominis* (right).

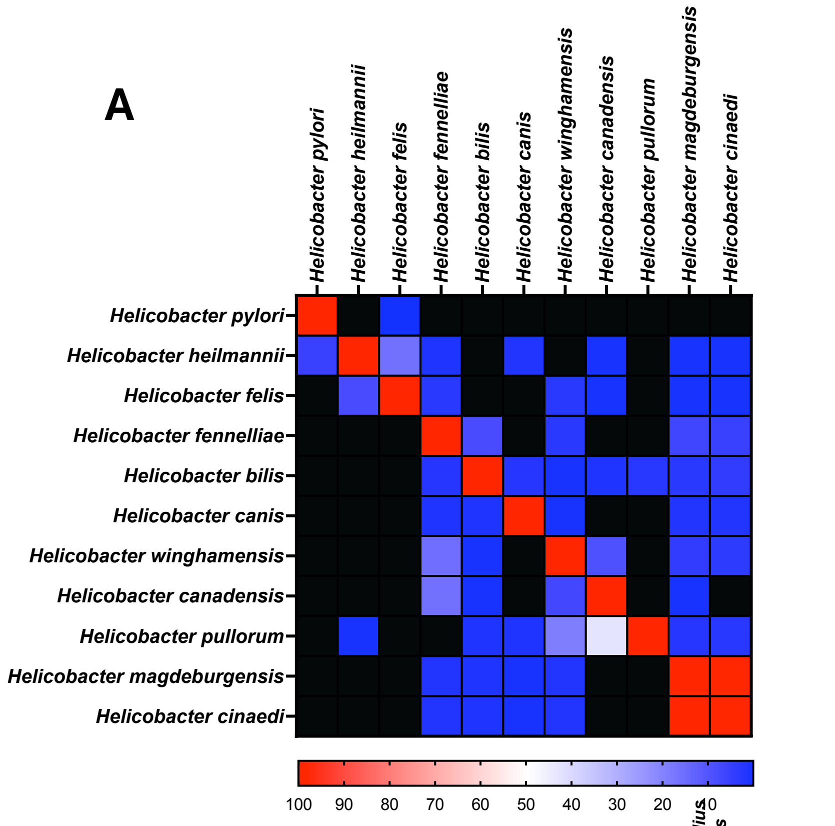

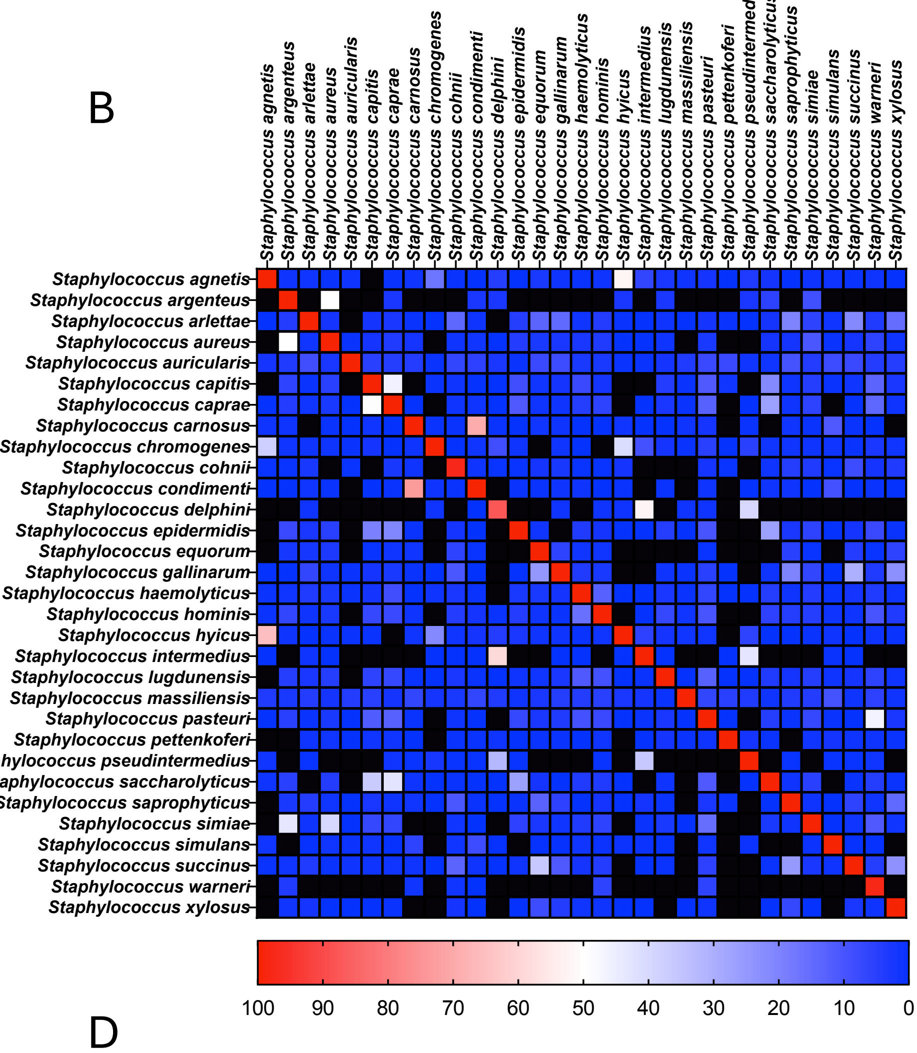

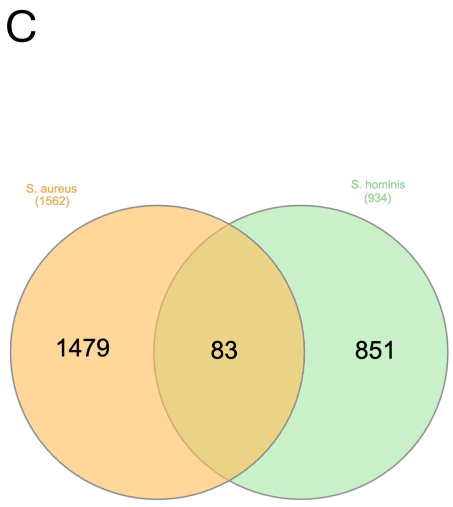

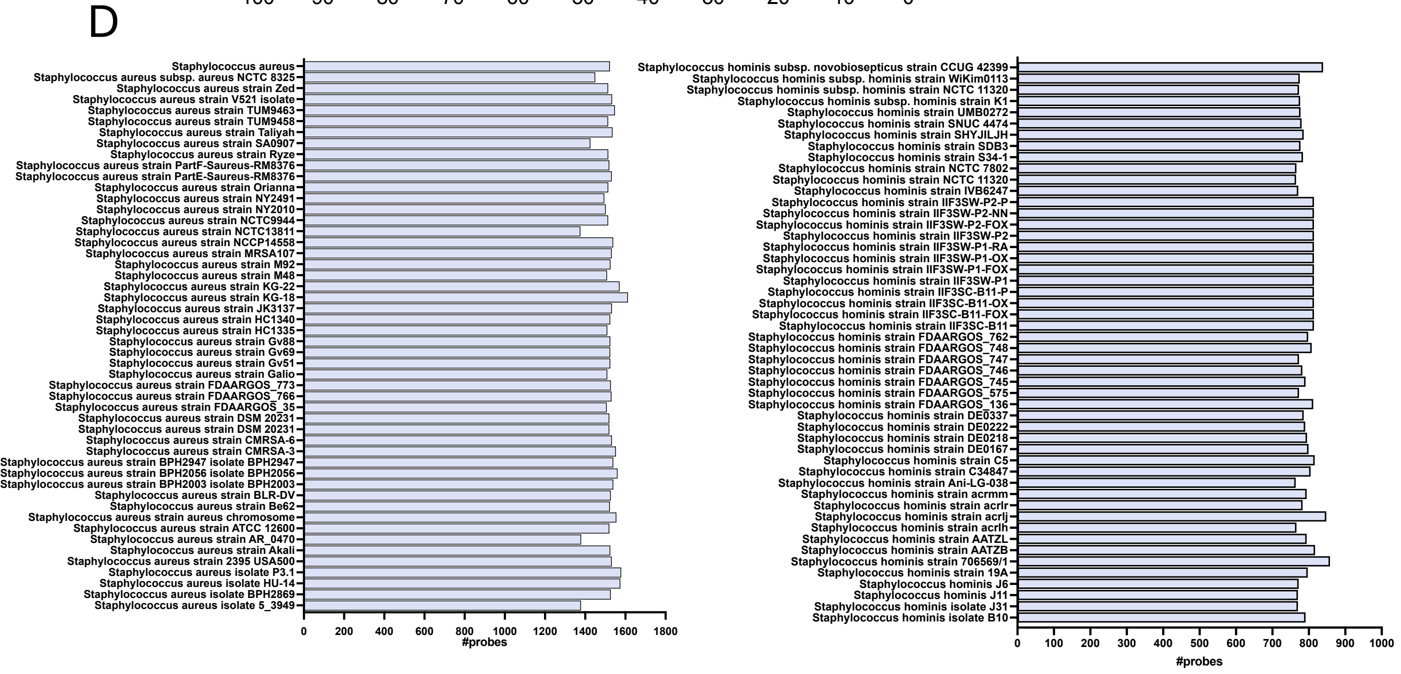

A

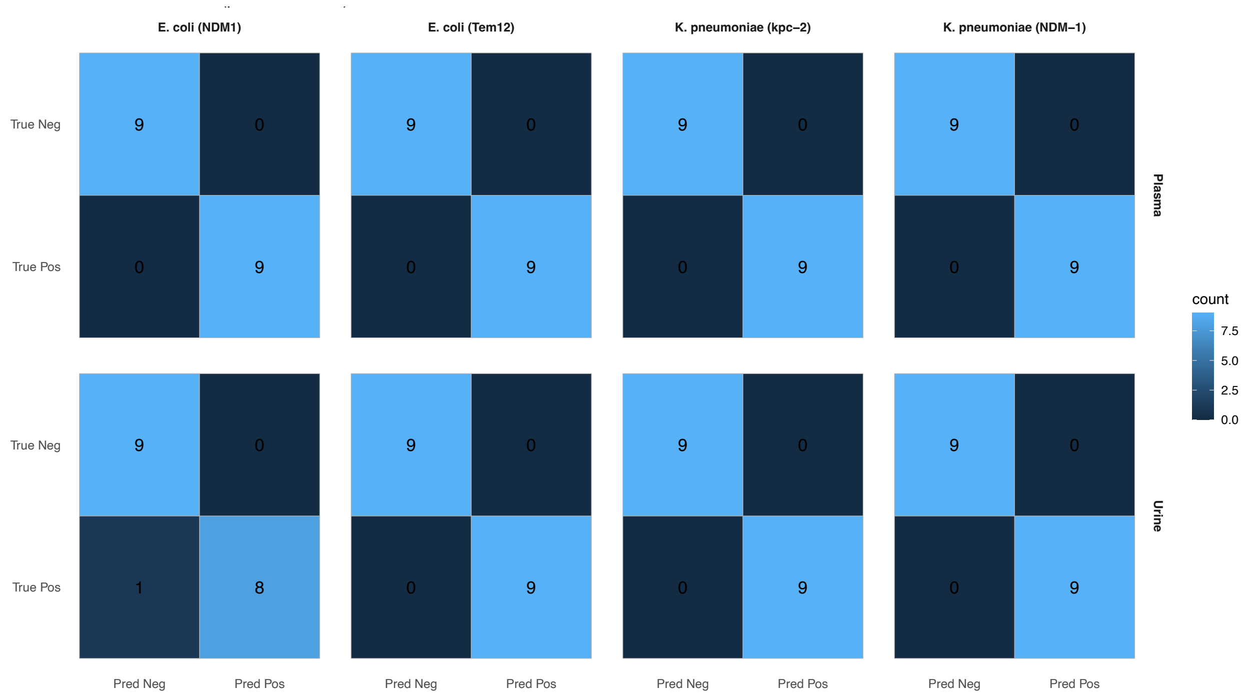

B

	E. coli (256922)		*E. coli (TEM-12)*		*E. coli (NDM-1)*		*K. pneumoniae (NDM-1)*		*K. pneumoniae (KPC-2)*	
	Plasma	Urine	Plasma	Urine	Plasma	Urine	Plasma	Urine	Plasma	Urine
Mean (log)	3.73	3.53	3.87	4.16	3.25	3.1	3.92	3.73	3.31	3.37
SD	0.33	0.72	0.3	0.25	0.25	0.63	0.22	0.9	0.54	0.35
SEM	0.11	0.24	0.1	0.08	0.08	0.21	0.07	0.3	0.18	0.12
CV (%)	8.78	20.33	7.77	6.12	7.77	20.46	5.58	24.11	16.18	10.4
	H. influenzae		*M. intracellulare*		*N. meningitidis*		*P. aeruginosa*		*P. mirabilis*	
	Plasma	Urine	Plasma	Urine	Plasma	Urine	Plasma	Urine	Plasma	Urine
Mean (log)	4.3	4.94	2.83	3.8	3.39	3.63	4.59	4.52	4.33	4.2
SD	1.01	0.23	1.12	0.71	1	0.1	0.23	0.13	0.26	0.25
SEM	0.34	0.08	0.37	0.24	0.33	0.03	0.08	0.04	0.09	0.08
CV (%)	23.4	4.58	39.6	18.66	29.52	2.81	4.96	2.88	6.05	5.92

Insilco validation of probe design. (**A**) Confusion matrix for resistance genotype identified in 9 replicates for bla*NDM-1* and bla*TEM12* in *E. coli* and bla*KPC-2* and bla*NDM-1* in *K. pneumoniae* in plasma and urine. (**B**) statistics representing the mean, standard deviation (SD), standard error of means (SEM) and coefficient of variation (CV) for 9 replicates for reproducibility and repeatability for identification of bacterial agents in plasma and urine.
