# Supplementary material for: BCS2.0: a capture sequencing platform for rapid differential diagnosis of bacterial infections and antimicrobial resistance
Source: Front Microbiol. 2026 Apr 20;17:1802485. doi: 10.3389/fmicb.2026.1802485 (PMC13136086; doi:10.3389/fmicb.2026.1802485)
Supplement: Supplementary file 3 [file Table_2.DOCX]

| **Species** | **CFU/ml** | **UHTS** | **BCS2.0** | **Enrichment** | **UHTS** | **BCS2.0** | **Enrichment** |
| --- | --- | --- | --- | --- | --- | --- | --- |
|  |  | Whole genome (reads/M raw reads) | | | Target bacteria (reads/M raw reads) | | |
| *Escherichia coli* | 10 | 0 | 2581.88 | - | 0.88 | 1318.10 | 1499 |
|  | 100 | 1.72 | 32469.32 | 18877.51 | 7.14 | 12031.44 | 1685 |
|  | 1000 | 1343.94 | 194203.23 | 144.50 | 72.65 | 69628.17 | 958 |
|  | 10000 | 42410.64 | 303077.87 | 7.15 | 729.73 | 111455.75 | 153 |
| *Staphylococcus aureus* | 10 | 0.08 | 3438.69 | 42983.63 | 0.80 | 2090.57 | 2615 |
|  | 100 | 1.56 | 38206.14 | 24491.12 | 13.38 | 18622.68 | 1392 |
|  | 1000 | 2240.87 | 215572.10 | 96.20 | 142.40 | 103437.37 | 726 |
|  | 10000 | 36723.35 | 347773.07 | 9.47 | 1558.13 | 169727.40 | 109 |
| *Streptococcus agalactiae* | 10 | 0 | 111.72 | - | 0 | 242.12 | - |
|  | 100 | 0 | 3478.70 | - | 1.05 | 2453.11 | 2329 |
|  | 1000 | 114.24 | 43380.28 | 379.73 | 10.80 | 26699.72 | 2472 |
|  | 10000 | 2180.54 | 297618.02 | 136.49 | 117.44 | 182519.00 | 1554 |

**Table S2**: Comparison of bacterial genomic and targeted bacterial reads after BacCapSeq2.0 enrichment and untargeted high throughput sequencing (UHTS)
